# Multi-State Analysis of the Impact of Childhood Starvation on the Healthy Life Expectancy of the Elderly in China

**DOI:** 10.3389/fpubh.2021.690645

**Published:** 2021-07-05

**Authors:** Huiling Dong, Chunjing Du, Bingyi Wu, Qunhong Wu

**Affiliations:** ^1^Department of Public Health, Weifang Medical University, Weifang, China; ^2^Department of Management, Weifang Medical University, Weifang, China; ^3^Department of Health Management, Harbin Medical University, Harbin, China

**Keywords:** the elderly, healthy life expectancy, risk transition probability, childhood starvation, life course

## Abstract

**Background:** Child malnutrition is not only common in developing countries but also an important issue faced by developed countries. This study aimed to explore the influence and degree of childhood starvation on the health of the elderly, which provides a reference for formulating health-related policies under the concept of full lifecycle health.

**Methods:** Based on the Chinese Longitudinal Healthy Longevity Survey (CLHLS) in 2008, 2011, and 2014, this study took a total of 13,185 elderly people aged 65–99 years as the target population. By IMaCH software, with gender and income level as the control variables, the average life expectancy and healthy life expectancy of the elderly were measured. The *x*^2^test was used to explore the differences in the socioeconomic status of elderly people with or without starvation in childhood. Statistical differences between average life expectancy and healthy life expectancy were analyzed by rank tests.

**Results:** (1) The results showed that there was a statistically significant difference in age, gender, residency, education level, and income level between the groups with or without starvation (*P* < 0.05). (2) Transition probabilities in health–disability, health–death, and disability–death all showed an upward trend with age (*P* < 0.05), where the elderly who experienced starvation in childhood were higher than those without such an experience (*P* < 0.05). However, the probability of disability–health recovery showed a downward trend with age (*P* < 0.05), in which the elderly who experienced starvation in childhood were lower than those without starvation (*P* < 0.05). (3) For the elderly who experienced starvation in childhood, the health indicators of the average life expectancy, healthy life expectancy, and healthy life expectancy proportion accounted for the remaining life were lower than those of the elderly without childhood starvation (*P* < 0.05).

**Conclusions:** The average life expectancy and healthy life expectancy of the elderly with childhood starvation are lower than those without childhood starvation. It shows that the negative impact of childhood starvation on health through the life course till old age has a persistent negative cumulative effect on the quantity and quality of life. Therefore, it is important to pay attention to the nutritional status of children in poor families from the perspective of social policymaking.

## Background

Since the 1900's, China has entered a violently turbulent modern society. During this period, the lives of people were generally difficult, and hunger caused a large number of deaths. After the establishment of the People's Republic of China in 1949, the country experienced a “three-year difficult period” from 1959 to 1961, which caused a nationwide “famine.” The number of deaths caused by starvation had risen, and the lack of nutrition greatly affected the health of the population. In recent years, some studies analyzed the impact of the “Great Famine” in childhood on the health and economic status of their adulthood and found that infants and children who had grown up in that period had shorter life longevity and poorer health in old age (1). The factors show that the health status of the elderly is not only affected by elements of the old stage, but also earlier life experiences, especially childhood.

Life course theory has gradually become an important paradigm for the study of elderly health, emphasizing the long-term impact of life events in critical periods on the health outcomes of the elderly. Existing research shows that childhood is a critical period of growth and development (2). At this time, negative nutrition, and health shocks experienced may change the original development track of the individual and thus affect the health trajectory of their entire life course. The elderly who experienced starvation during childhood as a health disadvantaged group have current disadvantages that may depend on the previous unfavorable socioeconomic status. From a policy perspective, it is necessary to understand the long-term effects of malnutrition in earlier life, because child malnutrition is not only common in developing countries, but also an important issue faced by developed countries. Data show that there are approximately 1 billion people in the world who are malnourished, including 140 million preschool children under the age of 5, which will lead to permanent damage to their physical and cognitive development and even death due to nutritional diseases (3).

In recent years, economics has begun to pay attention to the long-term effects of life experiences in the fetus or early childhood on health, education, and labor market conditions, especially the health and nutritional status before the age of 5 (4). These studies generally used negative external shocks as an identification event, such as war, famine, rainfall, flu, etc., (5–7). Schellenberg J A, Victora C G, and other studies of low-income or middle-income countries such as Brazil, India, and South Africa have found that malnourished children had shorter height in adulthood, fewer years of education, and diminished labor productivity (8). Epidemiology and health economics in China are increasingly concerned about the impact of childhood health and developmental status on their health in the adult period. Scholars such as Chen and Zhou (9) analyzed the long-term effects of China's “Great Famine” on the health of those who experienced famine. Studies found that babies and children born or raised during this period had a lower height, poorer health, and economic status in adulthood.

Although, there are many literatures on widely acknowledged links between childhood hunger and a range of adverse health outcomes late in life, the reliability of research results and research conclusions is different because of different measurement indicators. A comprehensive evaluation of the long-term impacts of hunger on an individual's health capital is empirically difficult to conduct. Earlier investigation of this issue was hindered by several challenges including data restrictions. To determine the long-term consequences, we need both information about whether a person experienced hunger several decades ago and information about health status. Data tracking individual experiences for such a long period are not often available even in developed countries (10, 11). The most frequent method in the previous literature is to use exposure to shocks defined at a more aggregated level (12), taking famine as an indicator of having childhood starvation (13–15). But the problem is that exposure to famine and exposure to hunger are not equivalent. Famine and hunger belong to different levels of variables. Therefore, identification strategies that only exploit macro-level variations may obtain inconsistent estimates of the long-term effects of hunger.

We deal with this problem by exploiting retrospective data on the individual-level occurrence of hunger episodes during childhood, collected by the Chinese Longitudinal Healthy Longevity Survey (CLHLS). This kind of measurement method is more effective, which can conduct a micro-analysis of how the dilemma in the early life is transformed into the negative results in later life. There is a growing literature taking advantage of this self-reported measure to examine the long-term consequences of childhood hunger associated with World War II and several famines that happened in European countries (16–18).

In summary, some researchers have done a lot of exploration in this field, but there are still many limitations. First, the above-mentioned studies have paid more attention to the lasting impact of severe nutrition and health shocks on the health of economically active people (people who have not yet reached old age). Second, in most of the previous research studies, self-assessed health status of the elderly was used as the dependent variable, by which body function of the elderly cannot be reflected effectively due to stronger subjectivity. As for the measurement of healthy life expectancy, most international literature adopts the multi-state life table method based on cohort data, which can reflect the true health level of the study population, and the research conclusions are more reliable (19, 20). Third, due to the lack of high-quality cohort data in China, most previous studies were based on cross-sectional data to measure the relationship between child hunger and health and cannot make statistical inferences. Therefore, this study takes life course theory as the analysis framework based on strict cohort data, whether to have the childhood starvation as independent variables, to measure healthy life expectancy of the elderly in China, trying to give an answer to the question: to what extent does the accumulated disadvantage formed by childhood starvation affect the health of the elderly?

## Methods

### Data

The data were derived from the CLHLS. This research project has conducted surveys eight times in 23 provinces in China. The project used the 2008 baseline data and the 2011 and 2014 follow-up data with a total of 13,185 people aged 65–99 years as samples. Since the survey did not select samples with equal probability, it was necessary to weigh the samples according to the actual composition of age, gender, and residence in the 23 provinces to represent the general population of the elderly in the country. Therefore, before calculating the relevant indicators, the study weighed the data according to the weight coefficients provided by the project team, after which the sample size was 16,200 people (based on the sixth census data of China) in order to better reflect the overall Chinese elderly population.

### Variables

The explained variable in this article is the health status of the elderly. The CLHLS database uses the ADL to determine the health status of the elderly. The scale includes six measurement items for eating, dressing, indoor activities, going to the toilet, bathing, and controlling toilet. For the above six measurement items, if the research subjects select “can be completed,” they are judged as “healthy”; if any one of the items is selected as “unable to complete,” the sample is judged as “disabled” (21). The question about income in the original questionnaire was, “Compared with the local people, what is your life?” There are five options. The options are set to “very rich,” “relatively rich,” “average,” “more difficult,” and “very difficult.” This study combined “very wealthy” and “relatively wealthy” into “rich” as a high-income group, and combined “average,” “relatively difficult,” and “very difficult” into “difficult” as a low-income group.

The core explanatory variable of this article is “Did you experience starvation in childhood,” to which the answer is “yes or no.” Childhood starvation in this study generally refers to physical hunger. Specifically, in childhood, due to food shortages, insufficient intake of energy and essential nutrients leads to changes in body structure and function. In the questionnaire of CLHLS, the exact age at which starvation occurred is not asked. Therefore, this article draws on the definition of children's physiological age in the Medicine, Education, and Labor Legislation field and defines childhood age from 0 to 18 years (22, 23).

### Preparation and Calculation of Multi-State Life Table

IMaCh software is used for the estimation of healthy life expectancy in this study, which is an abbreviation of Interpolated Markov Chain and one of the first batches of software to provide multi-state life table estimation. Its main advantage is the direct use of the original survey data, and the use of multi-period (≥2 times) different longitudinal data at intervals, in which processing different health statuses are considered such as improvement, reduction, no change, and death. In this study, State 1 and State 2 are healthy and disabled, respectively, and State 3 is dead.

In the multi-state life table, the initial state of the cohort (2008) is “healthy” and “unhealthy”; the end state (2014) is “healthy,” “unhealthy,” and “death.” Each state at the beginning of the period can be transited to any state at the end of the period. In this study, “whether childhood is starving” is defined as a binary variable, and “gender” and “income level” are included as control variables to calculate the multi-state healthy life expectancy of the elderly with or without starvation in childhood. The calculation formula of the main variables is as follows [Lievre et al. (24)]:

- Let X(x) denote the state of an individual aged x. After time h, this individual is in state X(x+h). Assume that X(x) is a non-homogeneous discrete parameter Markov chain on these three states with transition probabilities:
  (1) $$\begin{array}{r} {\;}_{h}p_{x}^{jk}=\mathrm{Pr}(X(x+h)=k/X(x)=j) \end{array}$$
- If this individual is observed only once more at time*t*_3_, and noted to be in state l, then a further contribution to the likelihood is ${(}_{d2}p_{x2}^{kl})$. In this case, the component of the total likelihood due to individual i is:
  (2) $$\begin{array}{r} L^{\left(i\right)}={(}_{d1}p_{x1}^{jk})\times {(}_{d2}p_{x2}^{kl}). \end{array}$$
  One observes that the formation of the likelihood is no trivial matter since there is no simple analytical expression for the higher-order transition probabilities.
- If θ denotes the vector of parameters and $\hat{\theta }$ its maximum-likelihood estimator, then standard theory tells that for a large sample of size N, the MLE$\hat{\theta }$ is approximatively normally distributed with mean θ and covariance matrix V ($\hat{\theta }$):
  (3) $$\begin{array}{r} lim_{N\to \infty }E\left(\hat{\theta }\right)\;=\;\theta \end{array}$$
  (4) $$\begin{array}{r} V\left(\hat{\theta }\right)=\frac{1}{N}I^{-1}\left(\theta \right) \end{array}$$
  Where, I(θ) is the information matrix computed at the true value θ. This implies the asymptotic normality of the estimates of the transition probabilities and health expectancies.
- The initial state was i. The proportion of outcome status was 1 (health) and 2 (disability). The prevalence ${\;}_{t}W^{i1}\left(x\right)$ among survivors at age x and in state 1 from a cohort of individuals in state i at age x - t (t years earlier) reads:
  (5) $$\begin{array}{r} {\;}_{t}w^{i1}(x)=\frac{{\;}_{t}p_{x-t}^{i1}}{{\;}_{t}p_{x-t}^{i1}{+}_{t}p_{x-t}^{i2}} \end{array}$$
  and the prevalence ${\;}_{t}W^{i2}\left(x\right)$:
  (6) $$\begin{array}{r} {\;}_{t}w^{i2}(x)=\frac{{\;}_{t}p_{x-t}^{i2}}{{\;}_{t}p_{x-t}^{i1}{+}_{t}p_{x-t}^{i2}} \end{array}$$
- Calculate the incidence rate of individual ending status j (the stable disability rate in the state j):
  (7) Py x.j(θ)=Py x1j(θ)+w2(x,θ)(Py x2j(θ)−Py x1j(θ))
- Over the interval (x, x + y), given the initial state i at age x, with y as the upper limit in the sums:
  (8) $$\begin{array}{r} {\;}_{y}e_{x}^{ij}\;=\;\sum_{u=1}^{y}uP_{x}^{ij} \end{array}$$
- Total life expectancies respective of the initial state are:
  (9) $$\begin{array}{r} e_{x}^{i.}\;=\;e_{x}^{i1}+e_{x}^{i2} \end{array}$$

### Statistical Analysis

First, the survey data were weighted using the weight coefficients provided by the CLHLS project team, and the weighted data were compared to the sixth census data of China from the age and sex, making the study sample more representative. Second, SPSS17.0 software was used to describe the frequency of different health statuses of the elderly. It analyzed the distribution of health status by age, gender, residency, education level, income level, and whether hungry or not. Third, *x*^2^ test was used to explore the differences in the socioeconomic status of elderly people with or without starvation in childhood, taking α = 0.05 as the inspection standard. Finally, by IMaCh software, the multi-state life table method was used to measure the average life expectancy and healthy life expectancy of the elderly people who have childhood starvation or not. Fourth, statistical differences between average life expectancy and healthy life expectancy were analyzed by rank tests.

## Results

### Data Quality Assessment

**Figure 1:**
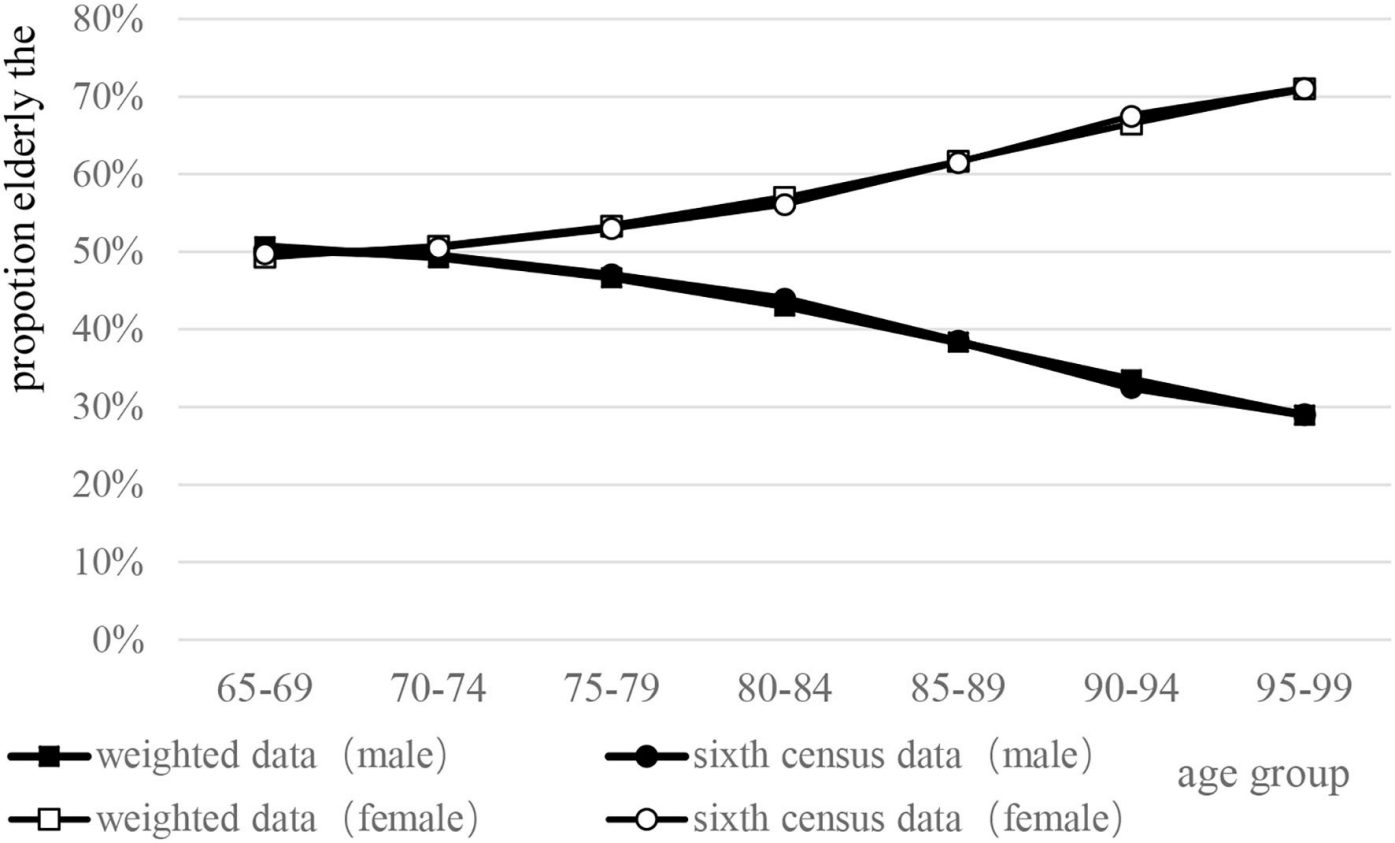
Comparison between the sixth census data of China and weighted survey data by sex–age structure.

Taking the sex–age structure of the elderly over 65 years in the sixth census in China as a reference, the data after weight adjustment of the cohort in the CLHLS database from 2008 to 2014 were compared. The results showed that the weighted adjusted data fitted well with the sixth census data of China (Figure 1).

### Descriptive Analysis

With the weighted adjustment of the raw data, the baseline number in 2008 was 16,200. The remaining number in 2011 was 14,405, and the number of survivors in 2014 was 12,876. In 2008, the proportion of elderly people under 80 years accounted for 83.51%. In 2011 and 2014, the proportion under 80 years old increased to 86.01 and 88.05%, respectively. The proportion of elderly females was higher than 50%, which was slightly higher than the elderly males. More than 60% of the elderly were farmers or unemployed; over 80% were primary and lower in education and lower income, which presented a declining trend over time. The proportion of elderly people with hunger in 2008 was 66.1%, rising to 65.9% in 2011 and 65.6% in 2014, respectively. From the perspective of health status, the proportion of healthy elderly decreased year by year during the follow-up period. Meanwhile, the proportion of disabled and dead elderly showed an upward trend (Table 1).

**Table 1:** Basic situation and health transition of the elderly.

	**2008 Year**		**2011 Year**		**2014 Year**	
**Explanatory variable**	***N***	**Proportion (%)**	***N***	**Proportion (%)**	***N***	**Proportion (%)**
**Age group**
65–69	5,590	34.51	5,351	37.16	5,097	39.59
70–74	4,696	28.99	4,266	29.62	3,870	30.06
75–79	3,242	20.01	2,770	19.23	2,369	18.40
80–84	1,755	10.83	1,401	9.73	1,109	8.61
85–89	696	4.30	495	3.44	352	2.73
90–94	184	1.14	106	0.74	66	0.51
95–99	37	0.23	16	0.11	13	0.10
**Gender**
Male	7,754	47.86	6,780	47.08	5,976	46.41
Female	8,447	52.14	7,622	52.93	6899	53.58
**Residency**
Urban	10,033	61.9	8,764	65.1	7,758	60.3
Rural	5,135	38.1	4,695	34.9	4,251	39.7
**Education level**
Primary schools and below	13,234	81.69	11,657	80.95	10,349	80.37
Above primary school	2,943	18.17	2,721	18.89	2,507	19.47
**Economic level**
Low income	14,115	87.1	12,507	86.85	11,136	86.49
High income	2,086	18.3	1,876	13.03	1,721	13.37
**Starvation**
Yes	10,709	66.1	9,489	65.9	8,442	65.6
No	5,492	33.9	4,912	34.1	4,434	34.4
**Health condition**
Health	15,405	95.09	10,331	63.77	8,024	49.53
Disability	796	4.91	1,393	8.60	4,627	28.56
Death	0	0	1,800	11.11	3325	20.52
Missing visits	0	0	2,677	16.52	225	1.39

### Single-Factor Analysis

The differences in the socioeconomic status of the two groups of elderly were explored. The results showed that there was a statistically significant difference in age, gender, residency, education level, and income level between the groups with or without starvation (*P* < 0.05). Specifically, the main features of the elderly with starvation experience were as follows: mainly over 80 years old, female (52.9%), rural (88.4%), lower education level with primary schools and below (91.9%), and mainly low income (87.8%). The detailed results are shown in the Supplementary Table 2.

### Risk Transition Probability

#### Health–Disability and Disability–Health Transition Probability

In general, the health–disability transition probability showed a linear upward trend with age [male: 95% CI (0.1244, 0.2715), female: 95% CI (0.1245, 0.2893), *P* < 0.05], whether male or female, whereas, the difference between the two groups gradually increased with age. On the contrary, the disability–health transition probability was linearly decreasing with age for all the elderly [male: 95% CI (0.094, 0.1716), female: 95% CI (0.0921, 0.1746), *P* < 0.05].

**Figure 2:**
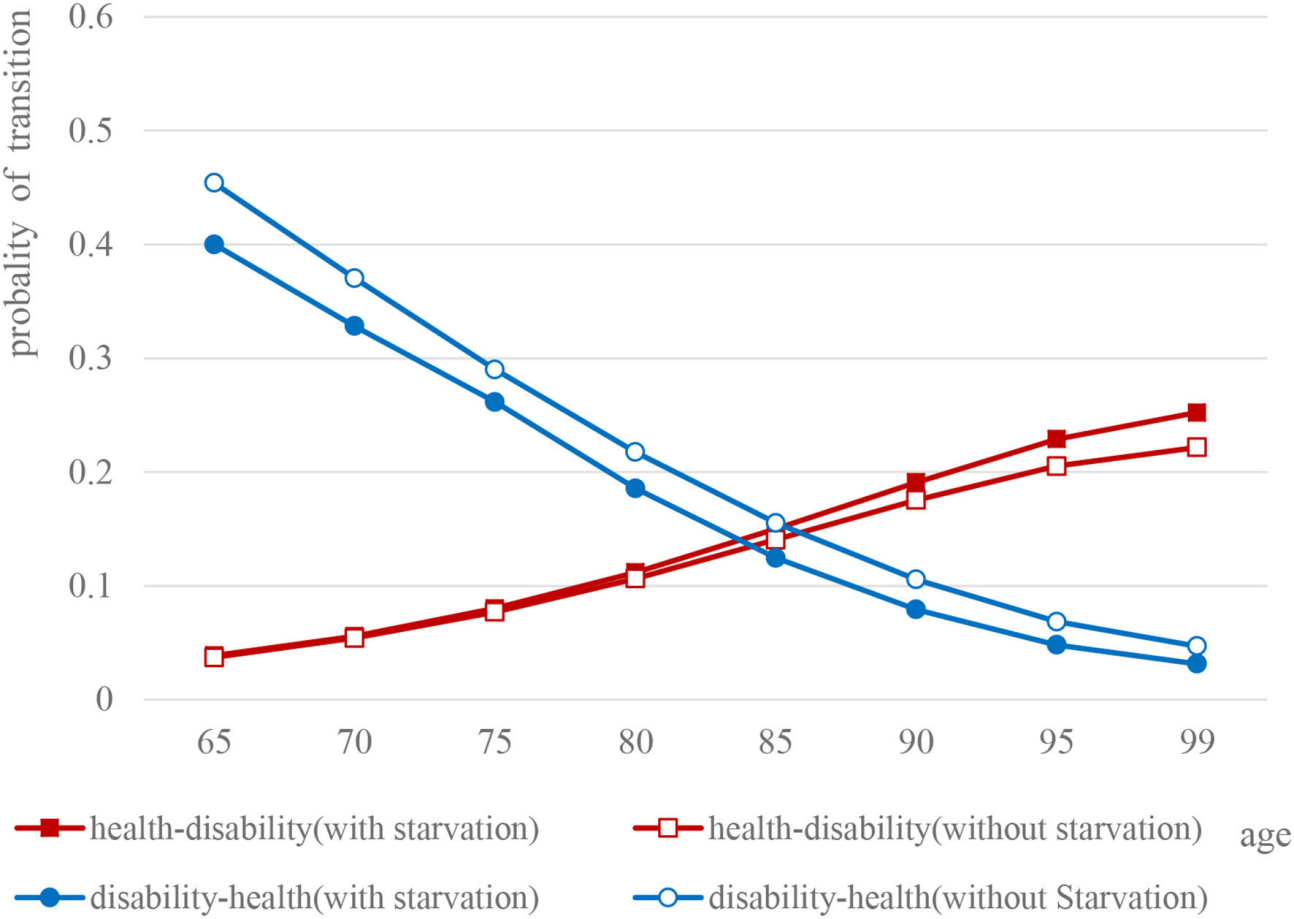
Comparison of the probability curves of disability and health transition among elderly males.

**Figure 3:**
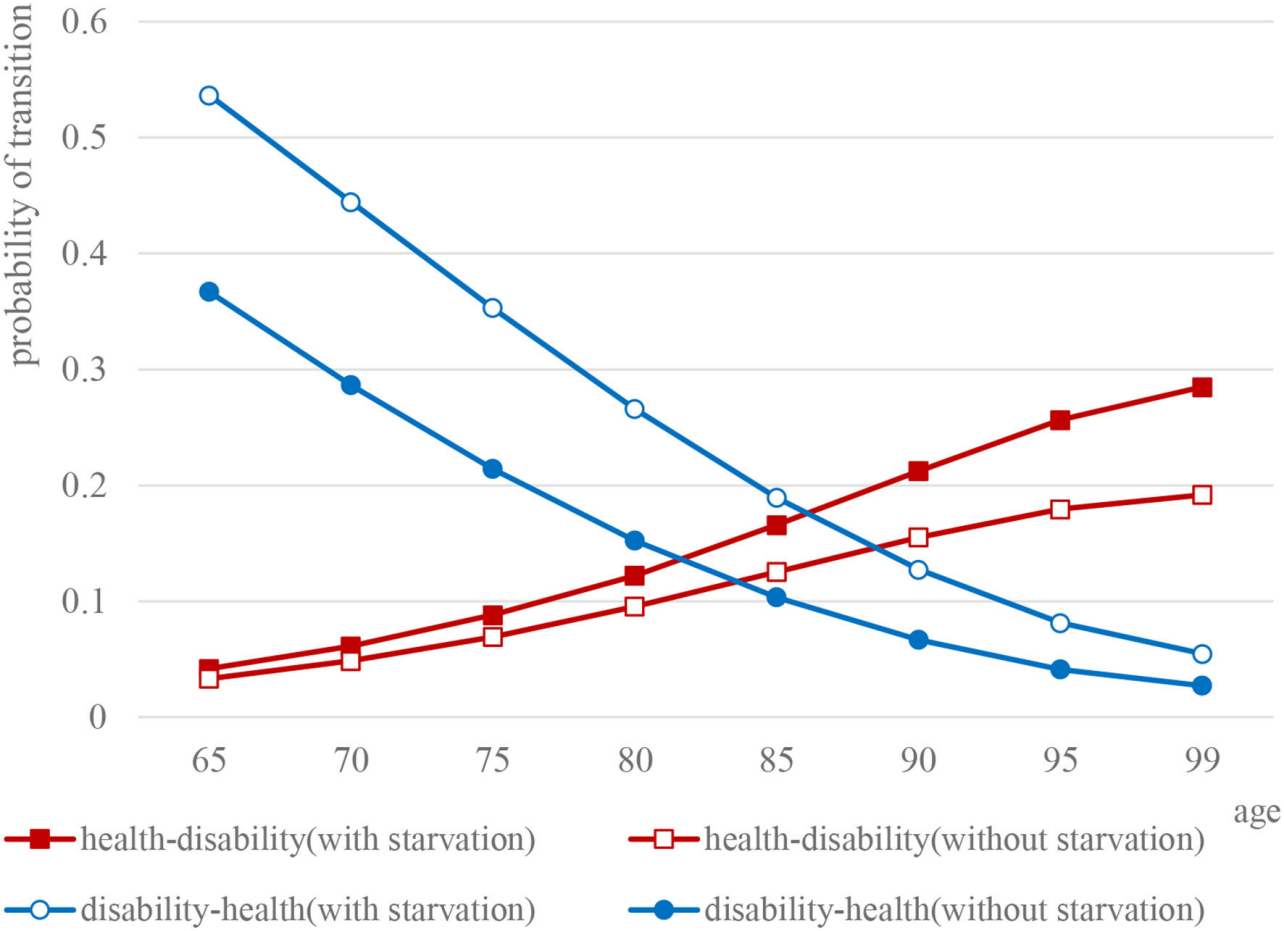
Comparison of the probability curves of disability and health transition among elderly females.

For the elderly males, the disability–health transition probability of the elderly who experienced starvation in childhood was lower than that of those without childhood starvation (*t* = 0.440, *P* < 0.05), especially for those over 80 years old. The difference between the two groups gradually widened with age. However, as for health–disability transition probability, the elderly who experienced starvation in childhood were higher than those without starvation (*t* = 0.526, *P* > 0.05). Elderly females (*t* = 3.279, *P* < 0.05) are similar to men. Meanwhile, for the probability of health–disability transition, the situation of the two groups was just the opposite (*t* = 0.999, *P* > 0.05) (Figures 2, 3).

#### Health–Death and Disability–Death Transition Probability

Overall, not only health–death [male: 95% CI (0.0988, 0.2480), female: 95% CI (0.0973, 0.2534), *P* < 0.05] but also disability–death transition probability [male: 95% CI (0.2516, 0.4789), female: 95% CI (0.2526, 0.4717), *P* < 0.05] of the elderly with or without starvation in childhood have both shown a linear upward trend, whereas, the difference between the two groups gradually expanded with age.

**Figure 4:**
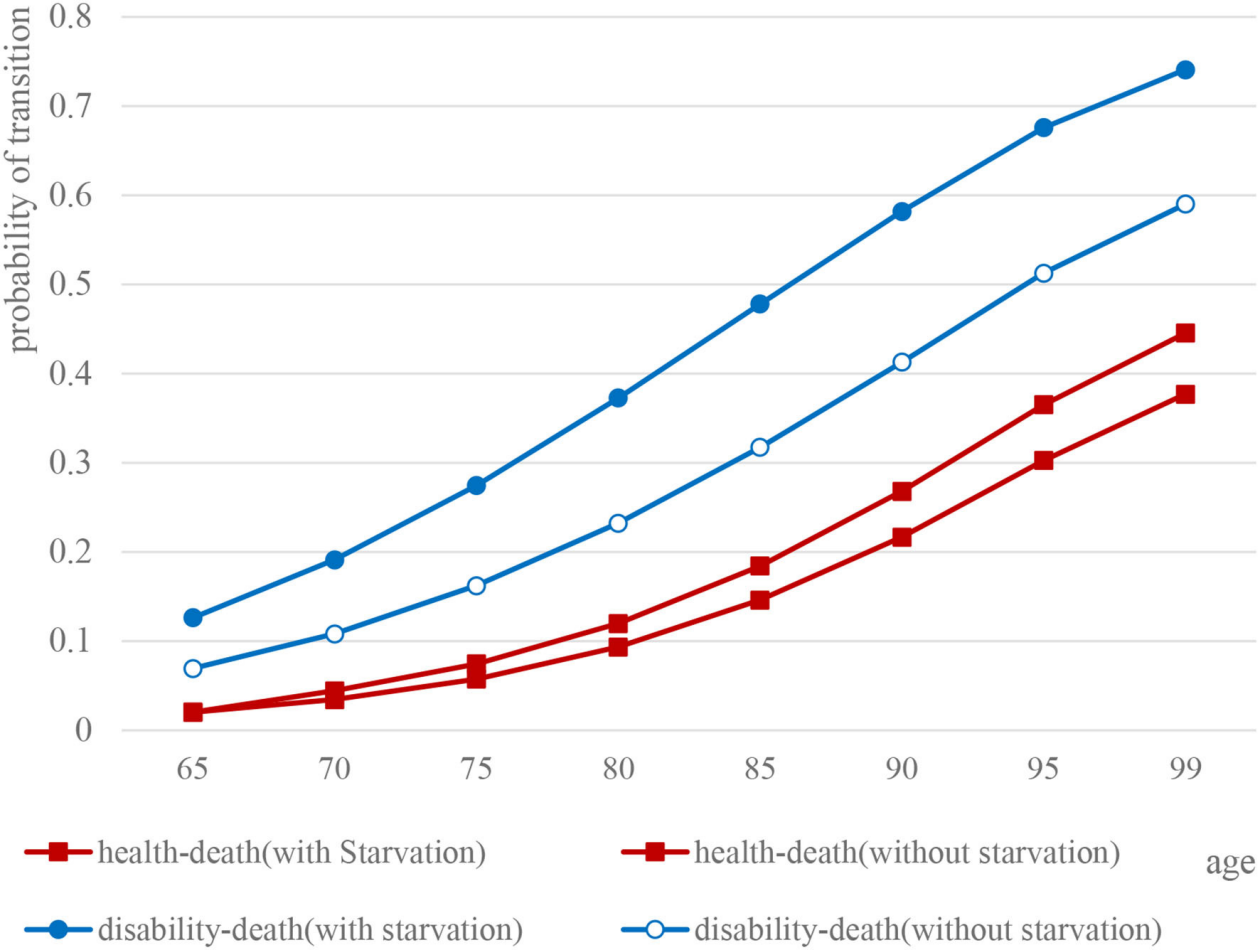
Comparison of health and death transition probability curves of elderly males.

**Figure 5:**
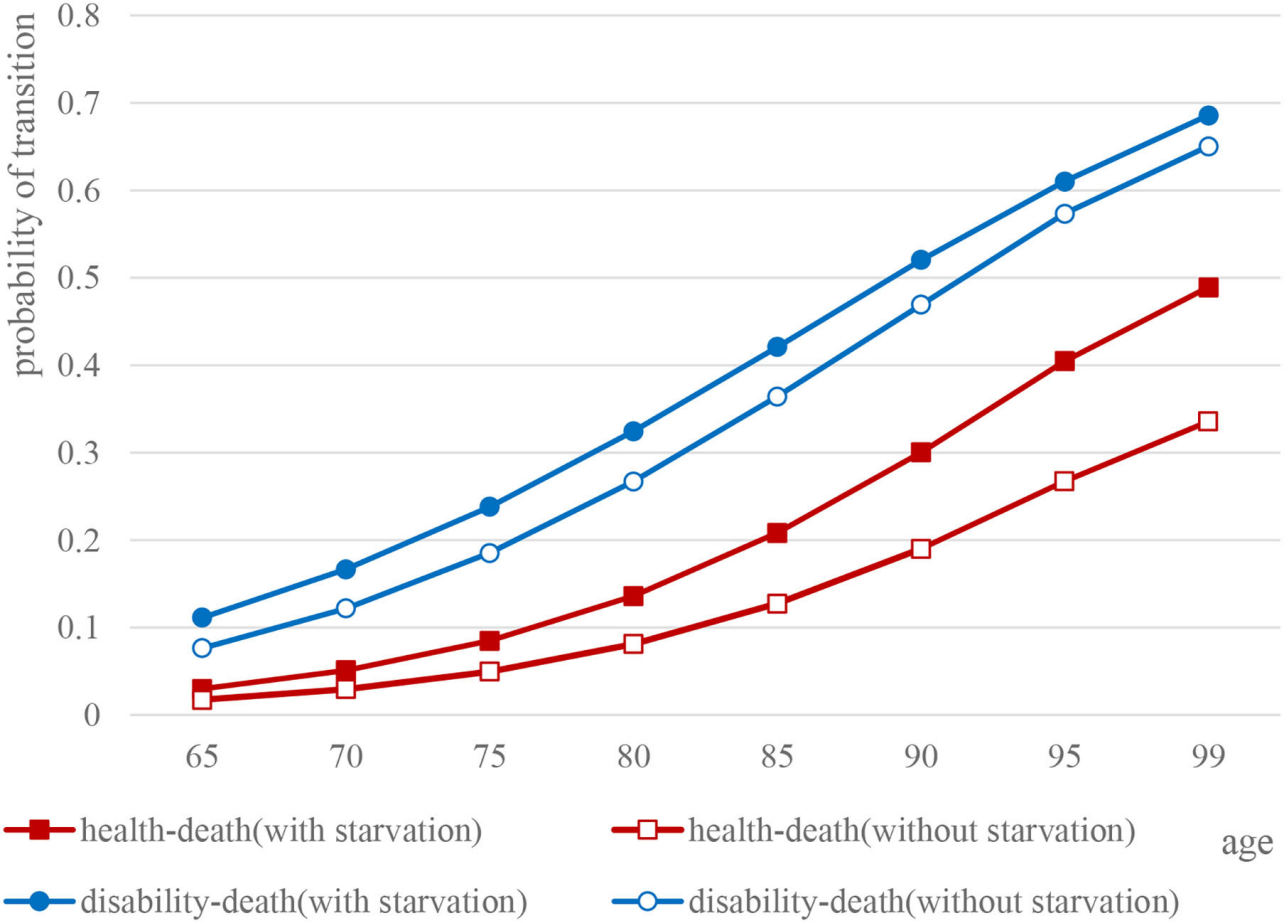
Comparison of health and death transition probability curves of elderly females.

Specifically, for elderly male people who experienced starvation in childhood, the probability of disability–death transition (*t* = 8.140, *P* < 0.05) and health–death transition probability (*t* = 2.079*, P* > 0.05) were both higher than for the elderly without experience of starvation. Similarly, as for the probability of both disability–death (*t* = 8.135, *P* < 0.05) and health–death (*t* = 1.873, *P* > 0.05), the elderly female who experienced starvation in childhood were higher than those without experience of starvation (Figures 4, 5).

### Analysis of Healthy Life Expectancy and Its Differences

Overall, regardless of male or female, the elderly who experienced starvation in childhood were lower than the elderly without starvation experience on such indicators as the average life expectancy, healthy life expectancy, and healthy life expectancy accounted for the remaining life, in which the difference between the two groups gradually decreasing with age on the average life expectancy [male: 95% CI (4.7241, 9.6559), female: 95% CI (5.8672, 11.6542), *P* < 0.05] and the healthy life expectancy [male: 95% CI (3.1915, 7.8079), female: 95% CI (3.7217,9.0226), *P* < 0.05], respectively.

For the elderly males, the HLE of the elderly between 65 and 69 years was 12.26 ± 0.26 years, while the LE was 14.36 ± 0.27 years, which meant that elderly males between 65 and 69 years were in a healthy state accounting for 85.30% of the time. In the same age group, the HLE of the elderly without hunger in childhood was 12.70 ± 0.21 years. Meanwhile, the LE was 14.78 ± 0.23 years, indicating that 65- to 69-year-old males without hunger had 85.92% in a healthy state for the rest of their lives. The paired *t*-test found that the HLE of the elderly without starvation in all age groups was higher than that of the elderly with starvation, with statistically significant difference (*P* < *0.05*), while the HLE/LE of the elderly without starvation was also higher than that of elderly people with starvation, with statistically significant difference (*P* < *0.05*).

For the elderly females, the HLE of those between 65 and 69 years with hunger experience was 14.06 ± 0.30 years, while the LE was 17.06 ± 0.33 years, which meant that the males between 65 and 59 years with hunger had 82.30% healthy state for the rest of their life. In the same age group, the HLE of the elderly without hunger in childhood was 14.48 ± 0.22 years. Meanwhile, the LE was 17.42 ± 0.25 years, indicating that the elderly males between 65 and 59 years without hunger had 83.1% in a healthy state of living (Table 2). The paired *t*-test found that the HLE of the elderly without starvation in all age groups was higher than that of the elderly with starvation, at which the difference was statistically significant (*P* < *0.05*). The proportion (HLE/LE) of the elderly without starvation was also higher than that of elderly people who experienced starvation, at which the difference was statistically significant (*P* < *0.05*).

**Table 2:** Comparison of healthy life expectancy among the elderly population (x ± s).

**Age group**	**With starvation**			**Without starvation**		
	**LE**	**HLE**	**HLE/LE (%)**	**LE**	**HLE**	**HLE/LE (%)**
**Man**
65–69	14.36 ± 0.27	12.26 ± 0.26	85.3	14.78 ± 0.23	12.70 ± 0.21	85.92
70–74	11.02 ± 0.24	9.03 ± 0.23	82.55	11.34 ± 0.19	9.39 ± 0.18	82.72
75–79	8.23 ± 0.21	6.36 ± 0.19	77.19	8.46 ± 0.17	6.65 ± 0.16	78.54
80–84	6.02 ± 0.18	4.30 ± 0.17	71.22	6.18 ± 0.24	4.53 ± 0.16	73.18
85–89	4.38 ± 0.16	2.80 ± 0.15	63.68	4.48 ± 0.14	2.99 ± 0.13	66.48
90–94	3.22 ± 0.13	1.77 ± 0.15	54.51	3.28 ± 0.12	1.92 ± 0.13	58.39
95–99	2.44 ± 0.11	1.08 ± 0.15	44.03	2.47 ± 0.09	1.22 ± 0.12	49.08
**Woman**
65–69	17.06 ± 0.33	14.06 ± 0.30	82.3	17.42 ± 0.25	14.48 ± 0.22	83.1
70–74	13.38 ± 0.30	10.51 ± 0.27	78.43	13.65 ± 0.22	10.87 ± 0.20	79.56
75–79	10.19 ± 0.38	7.50 ± 0.27	73.42	10.37 ± 0.20	7.79 ± 0.18	74.99
80–84	7.58 ± 0.24	5.09 ± 0.21	67.02	7.67 ± 0.19	5.32 ± 0.16	69.21
85–89	5.55 ± 0.22	3.29 ± 0.18	59.16	5.59 ± 0.17	3.48 ± 0.15	62.14
90–94	4.07 ± 0.19	2.04 ± 0.16	49.96	4.07 ± 0.15	2.20 ± 0.14	53.87
95–99	3.04 ± 0.16	1.22 ± 0.15	39.97	3.01 ± 0.13	1.36 ± 0.13	44.77

*①LE: average life expectancy; ②HLE: healthy life expectancy; ③HLE/LE: healthy life expectancy accounted for the remaining life*.

## Discussion

The life course provides an important theoretical perspective for a comprehensive analysis of the health status of the elderly. The results of this study showed that the experience of starvation in childhood had a negative cumulative effect on the health in old age, which was related to the social and historical environment of the research group. The target group was born in 1908–1942, which was a special historical period of social transformation, political turmoil, and material deprivation. During the time, many elderly people had experienced starvation before the age of 12. Some scholars have studied the long-term negative effects of “great famine of China” on the health of famine-experienced people (25–27). However, as a rare historical event, the “Great Famine” has serious, extreme, and transient characteristics, which conclusions drawn have certain limitations in terms of external validity. In contrast, the adverse effects of childhood starvation on health in this study are more typical and more universal.

The experience of starving in childhood affects the socioeconomic status of adulthood, which in turn affects the health outcomes of the elderly. The results of this study showed that the elderly who have experienced starvation in childhood were in rural (88.4%), mostly primary school and below in education (91.9%), and lower income level (87.8%). The literature that examined the long-term effects of fetal or childhood health as independent variables found that chronically poor health or malnutrition in childhood had a significant negative impact on the years of education during adulthood (28). Qing He and Yuan Yan analyzed the data of CHNS to show that the overall health status during childhood had a significant positive effect on adult income (29). Specifically, people with low socioeconomic status usually have cumulative disadvantages in terms of work environment, access to medical services, and health risks, which can affect their availability of health resources and health protection capacity (30).

The multi-state transition probability is the basis for measuring healthy life expectancy. When calculating the healthy life expectancy, the transitions between different multiple health states and the death risk could be taken to consideration, in which the result is closer to the health level of the crowd. This study found that regardless of the elderly with or without starvation in childhood, the transition probabilities showed an upward trend such as health–disability, health–death, and disability–death with age, while the probability of disability–health showed a downward trend. The elderly people with starvation in all age groups were lower than those who did not experience starvation in childhood. This result reflects that the impact of childhood nutrition on the health of people depends on the degree of hunger in childhood. Disability is a reversible state that can return to health, or it can lead to death. It means that we should pay more attention to the problem of malnutrition in childhood, earlier detection, earlier intervention, and earlier treatment, to not cause lasting adverse health effects.

The elderly who experienced starvation in childhood are lower than those without hunger in the three indicators, such as average life expectancy and healthy life expectancy, which result is closely related to the transition probability. Older people who experienced starvation in childhood had a higher probability of health–disability, but the probability of disability–health recovery was relatively low. Therefore, the probability of disability–health recovery is the key indicator to explain the difference between the two groups above. The lower health recovery rate may reflect lower utilization of medical services, on which the social status of education and economic status affect the conditions and quality of medical service utilization (31, 32). Therefore, good education and economic conditions can not only increase their utilization of health resources but also increase awareness of preventive healthcare, which can effectively reduce the possibility of disability and increase the rate of disability–health recovery (33). The elderly without childhood hunger has obvious advantages in this respect.

The policy enlightenment brought by the research is that the improvement of the material living standard in recent years, and the support of the social security system, cannot completely offset negative effects on the health status of the elderly with childhood starvation experience. Therefore, the government should strengthen nutrition and health interventions for poor children and effectively improve the nutrition and health status of children in poverty-stricken areas and families through the implementation of nutrition improvement programs for preschool children. At the same time, health investment on children is an important prerequisite for the elderly people bonus. At present, the delayed retirement age has taken shape in China, but the smooth implementation of this policy depends largely on the health of the elderly. The research in this article shows that the health problems of the elderly population should be considered from the perspective of the life course, and policymakers should have a forward-looking awareness to strengthen the nutritional improvement and health promotion of vulnerable groups in the early life.

First, due to the limitations of IMaCH model, if all the individual and family socioeconomic status and environmental variables were included as control variables, it was very difficult to calculate. Therefore, this study only took gender and age as the basic variable and the current income level as the control variable in the model. The second is that if the nutritional status in childhood is too bad and leads to death, then these people will not appear in the sample of 2008–2014. Therefore, the estimates obtained from the sample used in this article may have survivor bias, which will underestimate the impact of childhood starvation experience on the health of the elderly. Third, to explore the impact of childhood hunger on the health of the elderly, there will be a problem of recall bias. But the way to correct recall bias is to expand the sample size. The cohort data used in this study have a larger sample size of 13,185, which can greatly reduce the bias of recall on the research results.

## Conclusions

The negative impact of childhood starvation on health through life course till old age has a persistent negative cumulative effect on the elderly health. The average life expectancy and healthy life expectancy of the elderly with childhood starvation both are lower than those of the elderly without childhood starvation. This study meant that for the social groups with poor early nutritional status, the upward mobility of adult social class and the improvement of material living conditions can not completely offset the negative effects of the early hunger experience. Therefore, in order to achieve healthy aging, government decision-makers should have a sense of foresight and take systematic intervention of all factors affecting health from the early stage of life. The conclusions of this study are important to the comprehensive understanding of the elderly health impact mechanism and the evaluation of current child nutrition projects, such as child nutrition improvement projects in poor areas, nutrition improvement projects in preschool children, etc.,

## Data Availability Statement

The data used in this study are openly available in the Peking University open research data at: https://opendata.pku.edu.cn/dataset.xhtml?persistentId=doi:10.18170/DVN/XRV2WN.

## Ethics Statement

The studies involving human participants were reviewed and approved by Weifang Medical University. The patients/participants provided their written informed consent to participate in this study.

## Author Contributions

BW conceived and designed the study and contributed to materials/analysis tools. HD gathered and analyzed the data and wrote the paper. CD reviewed, edited, and approved the manuscript. All authors have read and approved the final manuscript.

## Conflict of Interest

The authors declare that the research was conducted in the absence of any commercial or financial relationships that could be construed as a potential conflict of interest.

## Abbreviations

LE: average life expectancy
HLE: healthy life expectancy
HLE/LE: healthy life expectancy accounted for the remaining life.
